# Weather, Built Environment, or Personal Factors: Predictors of Walking by Independent Living Residents With Frailty

**DOI:** 10.1093/geroni/igab046.1734

**Published:** 2021-12-17

**Authors:** Margaret Danilovich, Aura Espinoza, Christie Norrick

**Affiliations:** 1 CJE SeniorLife, Skokie, Illinois, United States; 2 CJE SeniorLife, Chicago, Illinois, United States

## Abstract

Environmental factors influence older adult physical activity. However, the evidence about which factors lead to increased physical activity is mixed and few have studied how these factors affect those with frailty or living in retirement communities. This study investigated how environmental and weather factors influence physical activity among pre-frail and frail older adults residing in independent living retirement communities. We used ActivPal accelerometers to measure 7-day step counts among (n=108) pre-frail and frail residents in 9 independent living residences in metropolitan Chicago. We conducted environmental audits using the MAPS Abbreviated tool and collected National Weather Service Station data (temperature, precipitation, and daylight minutes) during the ActivPal periods. Participants were on average 80.0 years, 74% female, and average daily step count was 3,450 (range 151 - 11,663). Four buildings were in suburban areas and 5 in urban areas and four were private-pay residences while 5 offered subsidized rent. ANOVA results showed private-pay buildings had higher total MAPS scores than subsidized buildings (p=0.001), and urban buildings had higher total MAPS scores than suburban buildings (p < 0.000). Mean step differences were non-significant between different building types: (mean steps = 3,317 private-pay, 3,629 subsidized, 3,536 urban, 3,350 suburban). Pearson product-moment correlations showed a positive association between steps and MAPS positive streetscape features (p=0.011). Multiple regression analysis showed higher temperature days, precipitation, and more minutes of daylight were associated with higher step counts (p=.04). Given the dramatic variation in individual step counts, future research should investigate personal factors that contribute to activity among independent living residents.

